# Supplementary material for: FERN – a Java framework for stochastic simulation and evaluation of reaction networks
Source: BMC Bioinformatics. 2008 Aug 29;9:356. doi: 10.1186/1471-2105-9-356 (PMC2553347; doi:10.1186/1471-2105-9-356)
Supplement: Additional file 1 — FERN distribution, Version 1.3. This archive contains the FERN source code and binaries as well as documentation and example models in FernML and SBML. [file 1471-2105-9-356-S1.zip › fern/doc/javadoc/fern/network/modification/ReversibleNetwork.html]

ReversibleNetwork


---


|  |  |  |
| --- | --- | --- |
| **Overview** \| **Package** \| **Class** \| **Use** \| **Tree** \| **Deprecated** \| **Index** \| **Help** | |  |
| **PREV CLASS**   NEXT CLASS | **FRAMES**    **NO FRAMES**     **All Classes** |  |
| SUMMARY: NESTED \| FIELD \| CONSTR \| METHOD | DETAIL: FIELD \| CONSTR \| METHOD |  |


---


## fern.network.modification Class ReversibleNetwork

```
java.lang.Object
  fern.network.modification.ModifierNetwork
      fern.network.modification.ReversibleNetwork
```

**All Implemented Interfaces:**: Network

---

``` public class ReversibleNetwork extends ModifierNetwork ```

Doubles each reaction in a way that each original unidirectional reaction becomes
reversible.As proposed by `ModifierNetwork`, the reactions are not copied
but the indices are redirected.

Note: The reverse reactions do not have any annotation, unless in the original network
the reactions have annotation with fields ending with `REVERSIBLE_SUFFIX`.
If such annotations are present in the original network, the corresponding reactions
in the reversible network will not have these annotations but the corresponding reversible
reactions will have these annotations without this suffix (e.g. if you create an `AutocatalyticNetwork`, the reactions have annotations `Catalyst` and  `CatalystReversible`, if you create a `ReversibleNetwork` out of this,
each reaction will have only the annotation `Catalyst`).

For the new reactions you have to specify a new `PropensityCalculator` whose index
space is equal to the original network's index space. If you have, for instance, in your
original network two reactions, A+A->B, B->C, the `PropensityCalculatory` has to
calculate the propensity for A+A<-B when index 0 is given, the propensity for B<-C, when
index 1 is given.

**Author:**
:   Florian Erhard

---

| **Field Summary** | |
| --- | --- |
| `static String` | `REVERSIBLE_SUFFIX`             Suffix which marks annotations for virtually created reverse reactions. |


**Constructor Summary**

`ReversibleNetwork(Network originalNet,
PropensityCalculator reversiblePropensityCalculator)`
  
          Creates a new network from an original network and virtually creates for each reaction a
new inverse reaction.


| **Method Summary** | |
| --- | --- |
| `AmountManager` | `getAmountManager()`             Gets the `AmountManager` for the modified network. |
| `AnnotationManager` | `getAnnotationManager()`             Gets the `AnnotationManager` for the modified network. |
| `int` | `getNumReactions()`             Gets the number of reactions in the modified network (which is 2\*number of reaction in the original network). |
| `int[]` | `getProducts(int reaction)`             Gets the products of a reaction |
| `PropensityCalculator` | `getPropensityCalculator()`             Gets the `PropensityCalculator` for the modified network. |
| `int[]` | `getReactants(int reaction)`             Gets the reactants of a reaction |
| `String` | `getReactionName(int index)`             Gets a string representation of the reaction in the modified network. |

**Methods inherited from class fern.network.modification.ModifierNetwork**

`getInitialAmount, getName, getNumSpecies, getOriginalNetwork, getParentNetwork, getSpeciesByName, getSpeciesName, setInitialAmount`

**Methods inherited from class java.lang.Object**

`clone, equals, finalize, getClass, hashCode, notify, notifyAll, toString, wait, wait, wait`

**Field Detail**

### REVERSIBLE\_SUFFIX

```
public static final String REVERSIBLE_SUFFIX
```

:   Suffix which marks annotations for virtually created reverse reactions.
    If there are no annotations with this suffix in the original network,
    the reverse reactions will have no annotations.

    **See Also:**: Constant Field Values


**Constructor Detail**

### ReversibleNetwork

```
public ReversibleNetwork(Network originalNet,
                         PropensityCalculator reversiblePropensityCalculator)
```

:   Creates a new network from an original network and virtually creates for each reaction a
    new inverse reaction. For the reverse reactions a `PropensityCalculator` has
    to be given.

    **Parameters:**: `originalNet` - the original network: `reversiblePropensityCalculator` - the `PropensityCalculator` for the reverse reactions


**Method Detail**

### getAmountManager

```
public AmountManager getAmountManager()
```

:   Gets the `AmountManager` for the modified network.

    :   **Specified by:**: `getAmountManager` in interface `Network` **Overrides:**: `getAmountManager` in class `ModifierNetwork`
    :   **Returns:**: `AmountManager` for the modified network

---


### getNumReactions

```
public int getNumReactions()
```

:   Gets the number of reactions in the modified network (which is 2\*number of reaction
    in the original network).

    :   **Specified by:**: `getNumReactions` in interface `Network` **Overrides:**: `getNumReactions` in class `ModifierNetwork`
    :   **Returns:**: number of reactions in the modified network

---


### getProducts

```
public int[] getProducts(int reaction)
```

:   Gets the products of a reaction

    :   **Specified by:**: `getProducts` in interface `Network` **Overrides:**: `getProducts` in class `ModifierNetwork`
    :   **Parameters:**: `reaction` - index of the reaction **Returns:**: indices of the products

---


### getReactants

```
public int[] getReactants(int reaction)
```

:   Gets the reactants of a reaction

    :   **Specified by:**: `getReactants` in interface `Network` **Overrides:**: `getReactants` in class `ModifierNetwork`
    :   **Parameters:**: `reaction` - index of the reaction **Returns:**: indices of the reactants

---


### getPropensityCalculator

```
public PropensityCalculator getPropensityCalculator()
```

:   Gets the `PropensityCalculator` for the modified network. If the
    original `PropensityCalculator` and the one for the reverse reactions
    is a `AbstractKineticConstantPropensityCalculator`, an `AbstractKineticConstantPropensityCalculator`
    is returned.

    :   **Specified by:**: `getPropensityCalculator` in interface `Network` **Overrides:**: `getPropensityCalculator` in class `ModifierNetwork`
    :   **Returns:**: the `PropensityCalculator` for the modified network

---


### getAnnotationManager

```
public AnnotationManager getAnnotationManager()
```

:   Gets the `AnnotationManager` for the modified network.

    :   **Specified by:**: `getAnnotationManager` in interface `Network` **Overrides:**: `getAnnotationManager` in class `ModifierNetwork`
    :   **Returns:**: `AnnotationManager` for the modified network.

---


### getReactionName

```
public String getReactionName(int index)
```

:   Gets a string representation of the reaction in the modified network.

    :   **Specified by:**: `getReactionName` in interface `Network` **Overrides:**: `getReactionName` in class `ModifierNetwork`
    :   **Parameters:**: `index` - index of the reaction **Returns:**: string representation of the reaction


---


|  |  |  |
| --- | --- | --- |
| **Overview** \| **Package** \| **Class** \| **Use** \| **Tree** \| **Deprecated** \| **Index** \| **Help** | |  |
| **PREV CLASS**   NEXT CLASS | **FRAMES**    **NO FRAMES**     **All Classes** |  |
| SUMMARY: NESTED \| FIELD \| CONSTR \| METHOD | DETAIL: FIELD \| CONSTR \| METHOD |  |


---
